# CDCA5-EEF1A1 interaction promotes progression of clear cell renal cell carcinoma by regulating mTOR signaling

**DOI:** 10.1186/s12935-024-03330-4

**Published:** 2024-04-24

**Authors:** Xun Wang, An Shi, Jie Liu, Wen Kong, Yiran Huang, Wei Xue, Fan Yang, Jiwei Huang

**Affiliations:** 1 Department of Urology, Renji Hospital Affiliated to Shanghai Jiaotong University School of Medicine, Shanghai, 200127 China; 2 Department of Critical Care Medicine, Renji Hospital Affiliated to Shanghai Jiaotong University School of Medicine, Shanghai, 200127 China; 3 Department of Pharmacy, Renji Hospital Affiliated to Shanghai Jiao Tong University School of Medicine, Shanghai, 200127 China

**Keywords:** CACD5, EEF1A1, Kidney cancer, Clear cell renal cell carcinoma, mTOR

## Abstract

**Background:**

Cell division cycle associated 5 (CDCA5) plays ontogenetic role in various human cancers. However, its specific function and regulatory mechanism in ccRCC remain uncertain.

**Methods:**

Immunohistochemistry and western blots were performed to investigate the expression of CDCA5 in ccRCC tissues. Genetic knockdown and upregulation of CDCA5 were performed to investigate its functional roles in ccRCC proliferation, migration, apoptosis and sunitinib resistance. Furthermore, Co-IP assay and LC–MS/MS were performed to investigate the underlying mechanisms.

**Results:**

We found that CDCA5 expression is frequently upregulated in ccRCC tumors and is associated with poor prognosis of ccRCC patients. Functionally, CDCA5 promotes proliferation, migration, and sunitinib resistance, while inhibiting apoptosis in ccRCC cells. In vivo mouse xenograft model confirms that silencing of CDCA5 drastically inhibits the growth of ccRCC. Mechanistically, we discovered that CDCA5 interacts with Eukaryotic Translation Elongation Factor 1 Alpha 1 (EEF1A1) to regulate mTOR signaling pathway, thereby promoting ccRCC progression.

**Conclusions:**

Taken together, our results demonstrate the significant role of CDCA5 in ccRCC progression. The findings may provide insights for the development of new treatment strategies targeting CDCA5 for ccRCC patients.

**Supplementary Information:**

The online version contains supplementary material available at 10.1186/s12935-024-03330-4.

## Background

Clear cell renal cell carcinoma (ccRCC), is the most common type of kidney cancer worldwide, originating from the renal tubular epithelial cells [1]. Clinically, surgery remains to be the mainstay for localized or early-stage ccRCC. However, some patients with radical nephrectomy may experience relapse and develop metastatic RCC [2]. Over the past two decades, various therapeutic approaches have emerged for advanced ccRCC, including immune therapies or molecularly targeted therapies that focus on particularly targeting receptor tyrosine kinase (RTK) or mammalian rapamycin (mTOR) [3]. However, long-term efficacy of these treatments is limited due to acquired drug resistance and severe side effects [4]. Hence, it is urgent to understand the underlying mechanisms of ccRCC development and identify new potential targets for ccRCC therapy.

CDCA5, also known as Sororin, is a key regulator for segregating sister chromatids during S and G2/M phases of the cell cycle [5]. CDCA5 protein maintains the cohesion of sister chromatids and ensures accurate chromosome separation during mitosis [6]. Recent studies have highlighted the significant role of CDCA5 in tumorigenesis and tumor progression. CDCA5 promotes cell proliferation and influences apoptosis by regulating the function of cell cycle related proteins and transcriptional factors [7]. Increased CDCA5 expression has been observed in various cancer types, including bladder cancer, lung cancer, liver cancer, colorectal cancer, indicating its potential involvement in the development of these diseases [8–11]. For example, CDCA5 activates PI3K/mTOR pathway, promoting proliferation and epithelial-mesenchymal transition (EMT) of breast cancer cells [12]. Additionally, CDCA5 depletion inhibits the proliferation and metastasis in esophageal squamous cell carcinoma [13]. CDCA5 is also a potential prognostic indicator in these tumors. However, the precise functional role of CDCA5 in ccRCC progression remains unclear.

In this study, we report that CDCA5 expression is overexpressed in ccRCC samples and patients with higher CDCA5 expression display poorer prognosis. Knockdown of CDCA5 inhibits the progression and tumorigenesis ability of ccRCC cells in vitro and in vivo. Mechanically, CDCA5 interacting with EEF1A1 to regulate mTOR signaling pathway, thereby promoting ccRCC progression. Collectively, our findings provide deeper insights into the functional role of CDCA5 in ccRCC and suggest it as a promising target for ccRCC therapy.

## Methods

### ccRCC patients and primary tissue samples

A total of 533 ccRCC patient cohort with Clinical Matrix and RNA sequencing data (HiSeqV2) were obtained from TCGA data portal. Patients with available prognosis data were selected when analyzing the relationship between CDCA5 expression and ccRCC progression. Besides, 20 paired tumorous and normal pericarcinomatous tissues were conserved in liquid nitrogen, which were collected from different pathological grades (WHO/ISUP 2016 grading system) of ccRCC patients undergoing nephrectomy at the Renji Hospital of Shanghai Jiaotong University. RT-qPCR, Western blot and immunohistochemistry (IHC) staining experiments were successively performed with these tissues. All patients provided written consent, and this study was approved by the Ethics and Research Committees of Renji Hospital, Shanghai Jiao Tong University School of Medicine, Shanghai, China.

### Cell culture and cell survival assay

ccRCC cell lines 786-O and ACHN were obtained from the ATCC (American Type Culture Collection) in 2020. 786-O cells were cultured in 1640 media with 10% fetal bovine serum (FBS, Gibco, Australia), and ACHN cells were cultured in MEM media with 10% FBS. All cells were maintained at 37 °C with 5% CO_2_. After treatment, cell survival was determined by MTT assay according to the standard protocol at different time points.

### Western blot analysis

Tissues or tumor cells were lysed with 2% sodium dodecyl sulfate (SDS) and the protein concentration was then measured with BCA protein assay kit. Next, proteins were separated by SDS-PAGE gel and transferred to a nitrocellulose membrane. After blocking, the membrane was incubated with different primary antibodies and secondary antibody (with washing between steps). Finally, the target protein bands were visualized via chemiluminescence system. Primary antibodies include: CDCA5 (ab192237) and eEF1A1 (ab157455) obtained from Abcam; p-AKT (Ser473) antibody (9271), p-S6 (Ser240/244) antibody (5364) and p-4EBP1 (Thr37/46) antibody (2855) were purchased from Cell Signaling Technology; β-actin (sc-69879) and GAPDH (sc-32233) were obtained from Santa Cruz. Secondary antibody Rabbit IgG (#7074) and Mouse IgG (#7076) were bought from Cell Signaling Technology.

### Immunohistochemistry

Immunohistochemistry was performed on tissue samples according to the standard streptavidin-peroxidase method. Briefly, after fixing, embedding, deparaffinizing, rehydrating and blocking, specimens were incubated with the anti-CDCA5 primary antibody (1:100 dilution) at 4 °C overnight. After washing and incubating with biotinylated secondary antibody, the results were finally recorded with a Nikon Eclipse Ti microscope. CDCA5 expression group was classified as follows: < median value, low expression, and ≥ median value, high expression.

### RNA extraction and RT-PCR

Trizol reagent was used to isolate total RNA from tumor samples and cells, and RNA was converted into cDNA with the cDNA synthesis kit according to the manufacturer’s protocol. Gene mRNA expression level was measured by RT-PCR on an ABI ViiA™ 7 System (Thermo Fisher, Waltham, MA, USA). GAPDH was used as the relative control for analyzing gene expression fold change. The following primers were used in this study: CDCA5, forward 5′-CCATCTCCTACTAAGCCTCTGC-3′, reverse 5′-GCCACGATCCTCTTTAAGACGAT-3′; GAPDH, forward 5′-TGACTTCAACAGCGACACCCA-3′, reverse 5′-CACCCTGTTGCTGTAGCCAAA-3′.

### Lentiviral vectors construction and transfection

Lentiviral particles were constructed according to previous protocol. Briefly, CDCA5 shRNA sequences or scrambled shRNA sequence were integrated into the GV358 backbone plasmid, which was obtained from GENE (Shanghai Genechem Co., Ltd.). Then, the shRNA plasmid, Helper1.0 plasmid and Helper2.0 plasmid were co-transfected into 293 T cells. Supernatant of treated 293 T cells was collected and filtered at 48–72 h after transfection. Besides, the plasmids using Ubi-3FLAG(sigma)-MCS-SV40-puromycin to construct overexpression EEF1A1 lentiviral vectors. Flag-CDCA5 plasmid (CDCA5 OE) was also obtained from GENE. Tumor cells were infected with lentiviral particles in the presence of 10 μg/mL Polybrene regent. After treating media with puromycin for 2 weeks, ccRCC cells were collected for subsequent experiments.

### Flow cytometry analysis

Cell apoptosis analysis was performed by using annexin V and propidium iodide double supravital stain. Briefly, cells were collected, fixed, stained and measured by flow cytometry. Results were analyzed by using ModFit software according to the manufacturer’s instructions.

### Cell migration analysis

To investigate the effect of CDCA5 on ccRCC metastasis, cell wound healing and cell transwell migration test were performed. For cell wound healing assay, cells after treatment were plated in 6-well plates with over 90% confluence, and a sterile 100 μL pipette tip was used to scrape across cells. At special time points, cells that migrated into the wounded area were measured by a microscope. For cell transwell migration assay, the special transwell plates were used according the conventional protocol. Briefly, appropriate number of cells were collected and added to the transwell plate, and cells were cultured for 24 h. Then, cells were fixed, stained and dried, and five view fields cells number were counted for each sample with a microscope. The mean value was used as the migratory cell number.

### Tumor models

All animal studies have been approved by the Experimental Animal Ethics Committee of Shanghai Jiao Tong University (Shanghai, China). 4 to 6-week-old female nude mice (BALB/c nu/nu) were used for establishing the ccRCC tumor xenograft models. After infecting with lentiviral particles, around 5 × 10^6^ ACHN cells were collected and subcutaneously injected into the flank region of mice. When the tumors reached approximately 200 mm^3^, tumor volumes (V = length × width^2^/2) and body weights were measured twice a week using a caliper and an iron-balance. At the end of experiments, all the mice were sacrificed and the tumors were harvested, photographed, weighed, and saved in liquid nitrogen.

### LC–MS/MS analysis

Liquid chromatography–tandem mass spectrometry (LC–MS/MS) analysis was performed to screen out the potential interacting proteins of CDCA5. Firstly, ACHN cells were overexpressed with FLAG-CDCA5, and then cell lysates were collected in lysis buffer on ice. After centrifugation, the supernatants were incubated with an anti-Flag or anti-IgG antibody. Secondly, protein FLAG-beads were added to the supernatants, and proteins were eluted and subjected to SDS-PAGE electrophoresis. After protein digestion and peptide selection, LC–MS analysis was performed with the PD/MASCOT software. Finally, we conducted PPI (protein–protein interaction) network analysis to select the potential interacting proteins. Besides, coimmunoprecipitation (Co-IP) experiments were performed to confirm our findings.

### Statistical analysis

CDCA5 expression levels in different groups were compared by using the student’s t test, and the Kaplan–Meier method with log-rank test was plotted to analyze the overall survival curves. All experiments were performed over three times, and data were presented as mean ± SD. Significant differences between values obtained from different groups were determined using the two-tailed student’s t-test analysis. All statistical analyses were performed using Graphpad Prism 7.0, and P < 0.05 was considered statistically significant.

## Results

### CDCA5 expression correlates with ccRCC progression in patients

**Fig. 1:**
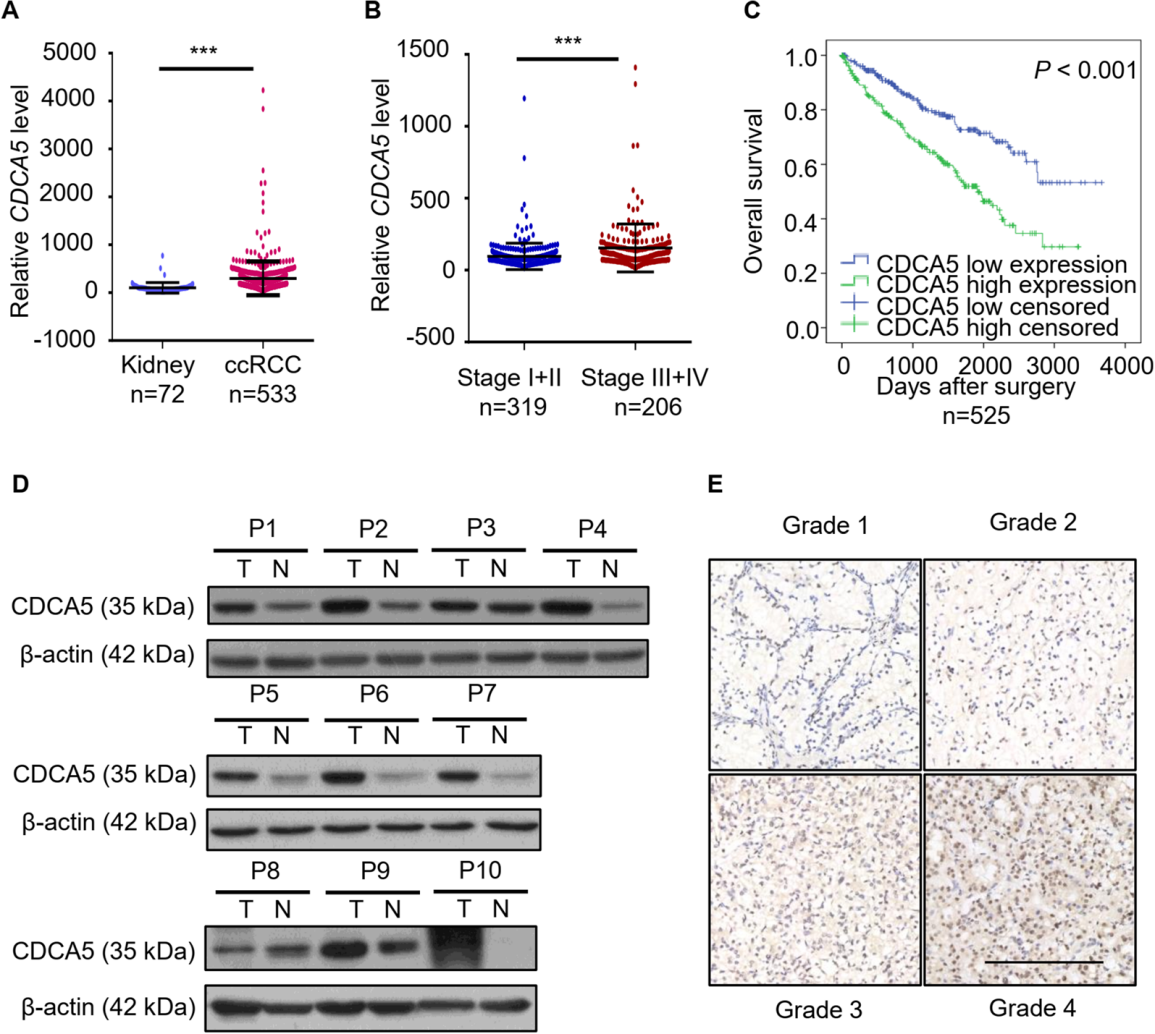
CDCA5 expression correlates with ccRCC progression in patients. **A, B** Data form TCGA dataset were analyzed to evaluate CDCA5 mRNA levels in normal kidney and tumor tissues (**A**), or different clinical stages (**B**). **C** Overall survival of patients stratified according to tumor CDCA5 expression. **D** Western blot analysis of CDCA5 protein expression in 10 ccRCC patients (T, tumor tissue; N, paired normal tissue). **E** Representative images of CDCA5 in different histological grades of ccRCC tumor tissues (n = 3 in each group). Scale bar: 100 um. ***P < 0.001

To assess the clinical significance of CDCA5 in renal clear cell carcinoma (KIRC), we firstly analyze clinical sample data deposited in Timer database (https://cistrome.shinyapps.io/timer/). Compared with normal tissues, CDCA5 is upregulated in various tumor types, such as in KIRC with statistical significance (Additional file 1: Fig. S1A). Five-year survival rate is higher in patients with lower expression of CDCA5 (Additional file 1: Fig. S1B). In line with this, TCGA database revealed increased CDCA5 gene expression in KIRC in comparison with normal kidney (Fig. 1A). In addition, CDCA5 is correlated with the malignant progression and poor prognosis of ccRCC patients (Fig. 1B, C). Examination of ccRCC tumor tissues and paired normal tissues (n = 10) by RT-qPCR and WB confirmed that the expression of CACD5 is remarkably increased in tumors (Fig. 1D, Additional file 1: Fig. S1C–D). To further investigate its clinical relevance in ccRCC, IHC analysis was performed in different grade tumor tissues (n = 12). Consistent with bioinformatics analysis, CDCA5 expression levels were significantly upregulated with increased pathological grade (Fig. 1E, Additional file 1: Fig. S1E). Taken together, these findings suggest that increased CDCA5 expression correlates with ccRCC progression. CDCA5 may function as a prognostic marker for ccRCC.

### Knockdown of CDCA5 inhibits ccRCC cell proliferation and migration in vitro

**Fig. 2:**
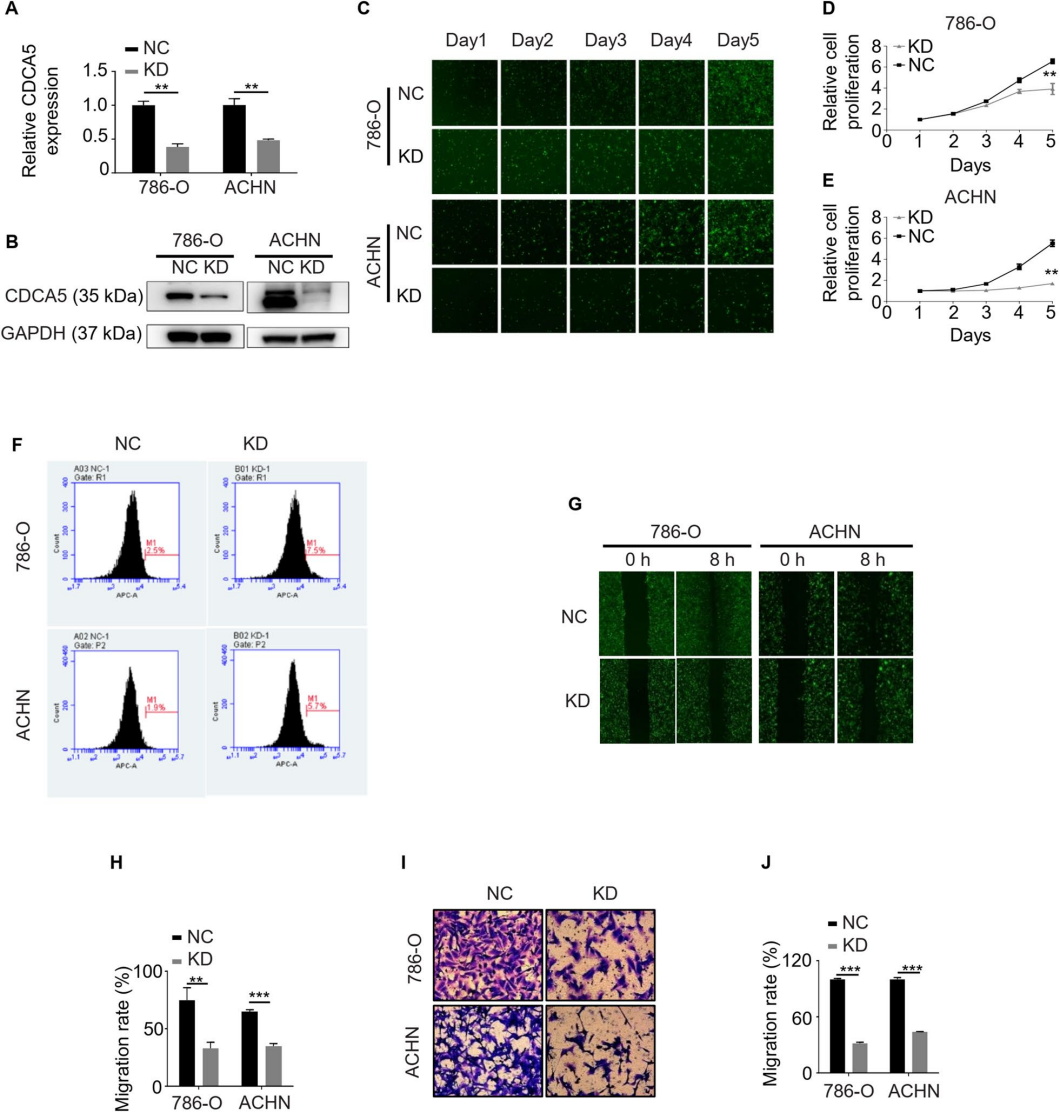
Knockdown of CDCA5 inhibits the proliferation and migration ability of ccRCC cells in vitro*.* **A, B** RT-qPCR (**A**) and WB (**B**) analyses of CDCA5 protein expression when knocking down of CDCA5 through lentivirus (CDCA5 shRNA). NC, negative control; KD, CDCA5 knockdown. **C** Cell survival assay with a fluorescence microscope in 786-O or ACHN cells with or without CDCA5 KD at indicated time points. **D, E** MTT analysis of cell proliferation in 786-O (**D**) or ACHN (**E**) cells with or without CDCA5 KD. **F** Annexin V and propidium iodide staining was performed to evaluated cell apoptosis of 786-O cells, 786-O cells with CDCA5 KD, as well as ACHN cells, ACHN cells with CDCA5 KD. **G–J** Wound healing (**G, H**) and cell migration (**I, J**) experiments to evaluate the effect of CDCA5 on 786-O and ACHN cell metastasis in vitro. **P < 0.01 and ***P < 0.001. NC, negative control; KD knockdown

To investigate the functional role of CDCA5 in ccRCC progression, CDCA5-specific knockdown models were developed in ccRCC cells, 786-O and ACHN. The knockdown efficiencies were confirmed by RT-qPCR and WB experiments (Fig. 2A, B). Fluorescence microscope (Celigo) and MTT analysis demonstrated that knockdown of CDCA5 significantly attenuated the proliferation of 786-O and ACHN (Fig. 2C, E). To investigate the underlying mechanism through which CDCA5 influences cell proliferation, the effect of CDCA5 on cell apoptosis was determined using flow cytometry. The results indicated that knockdown of CDCA5 could induce apoptosis of 786-O and ACHN cells (Fig. 2F and Additional file 2: Fig. S2A). Caspases 3/7 are the central players in the process of apoptosis [14]. Therefore, we examined Caspase3/7 activity, and found that CDCA5 knockdown cells have higher Caspase3/7 activity (Additional file 2: Fig. S2B).

To investigate the effect of CDCA5 on tumor metastasis, wound healing and migration ability of ccRCC cells were further evaluated. The results indicated that CDCA5 knockdown significantly inhibited both wound healing and migration ability of 786-O and ACHN cells (Fig. 2G–J). Taken together, our data suggested that CDCA5 plays a critical role in the proliferation and migration ability of ccRCC cells in vitro.

### CDCA5 is involved in chemo-sensitivity of ccRCC cells to sunitinib

**Fig. 3:**
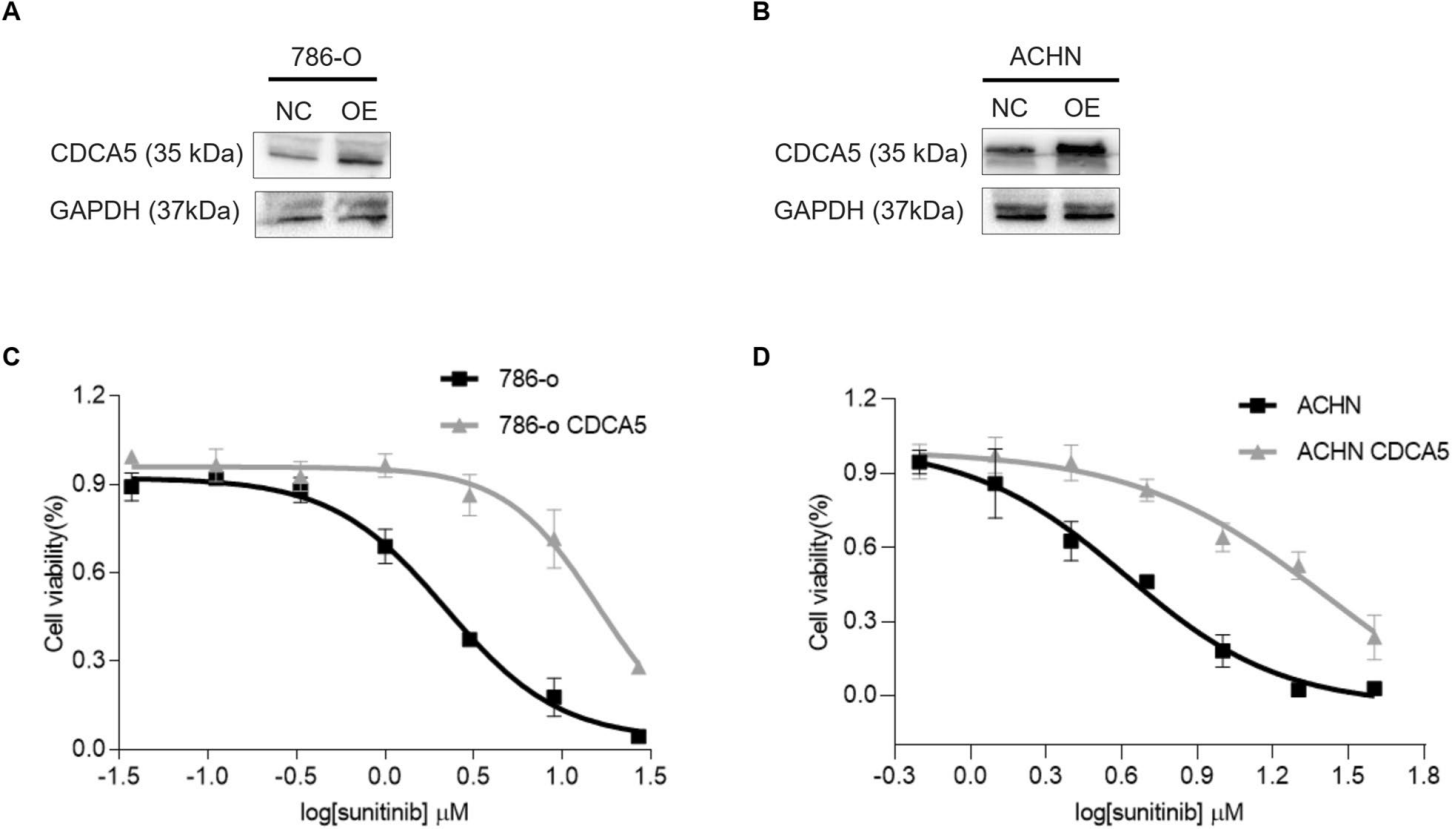
CDCA5 is involved in chemo-sensitivity of ccRCC cells to sunitinib. **A, B** WB analysis of CDCA5 expression when overexpressing CDCA5 (OE) in 786-O (**A**) or ACHN (**B**) cells. **C, D** Cell proliferation was examined using MTT assays after exposure to Sunitinib for 72 h in 786-O NC, 786-O OE and ACHN NC, ACHN OE cells. Data show mean ± SD from three biological replicates

Sunitinib is the first-line chemotherapy agent for metastatic ccRCC in clinical. However, the inherent and acquired resistance compromised its effectiveness [15]. Given that CDCA5 is reported to be associated with chemosensitivity in esophageal squamous cell carcinoma [13], we thus examined the influence of CDCA5 to sunitinib treatment. CDCA5 was introduced into 786-O and ACHN cells. Western Blot analysis indicated the increased CDCA5 expression (Fig. 3A, B). Sunitinib effectively inhibited the cell proliferation of control 786-O and ACHN cells in MTT assay, while CDCA5 overexpression (OE) dampened this effect (Fig. 3C, D), indicating that overexpression of CDCA5 promotes the chemosensitivity of ccRCC cells to sunitinib.

### CDCA5 knockdown antagonizes ccRCC tumor growth in vivo

**Fig. 4:**
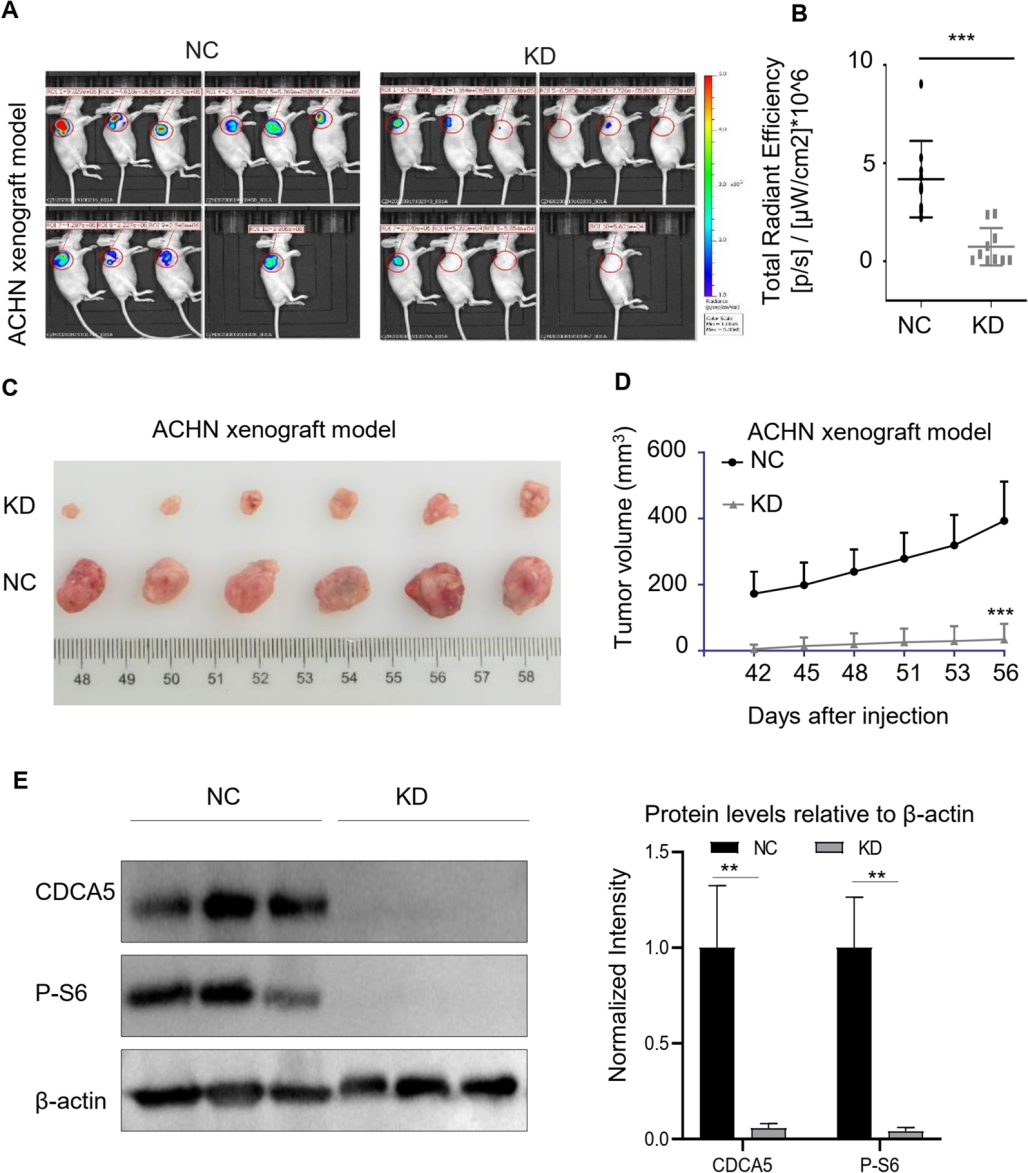
CDCA5 knockdown antagonizes ccRCC tumor growth in vivo. **A, B** ACHN cells (NC) or ACHN cells with CDCA5 knockdown (KD) were transplanted into nude mice. Tumor Growth was evaluated with the in vivo bioluminescence imaging system. **C, D** The representative photographs of ACHN tumor tissues in NC and KD groups (**C**), and tumor growth curves (**D**). N = 6 for each group. **E** WB analysis of P-S6, CDCA5 and β-actin levels in tumors (n = 3 in each group). The expression intensity was compared using arbitrary ratios normalized against β-actin. **P < 0.01 and ***P < 0.001

Having explored the tumorigenic role of CDCA5 in ccRCC in vitro, we next evaluated the anti-proliferative effect of CDCA5 knockdown in vivo using ACHN cells stably infected with lentiviruses expression negative control (NC) or CDCA5 knockdown (KD). These cells were subcutaneously injected into the flanks of nude mice, and the tumor growth was monitored twice a week. The results demonstrated that CDCA5 KD group showed a significantly reduced tumor growth, compared to the NC group. This was evident from reduced bioluminescence, tumor volume and tumor weight. (Fig. 4A–D and Additional file 3: Fig. S3A). Importantly, the reduction of tumor growth did not impact the bodyweight of the mice (Additional file 3: Fig. S3B). These findings provide strong evidence supporting the role of CDCA45 in promoting ccRCC growth in vivo. The inhibition of CDCA45 expression led to a significant reduction in tumor growth, indicating the potential of CDCA45 as a therapeutic target for ccRCC treatment.

### PI3K/AKT/mTOR signaling is essential for CDCA5 mediated ccRCC cell proliferation

**Fig. 5:**
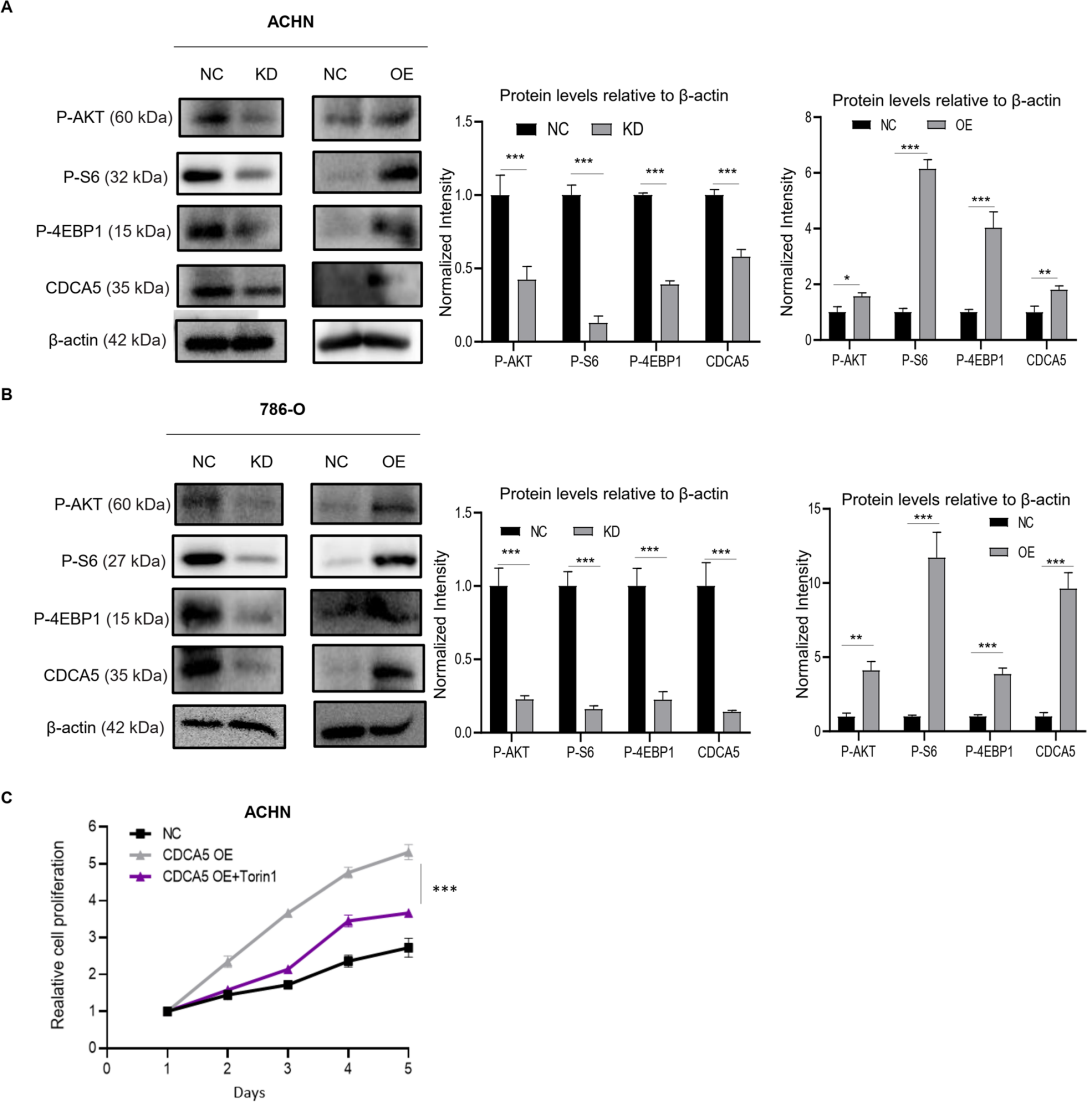
PI3K/AKT/mTOR signaling is essential for CDCA5 mediated ccRCC cell proliferation. **A**, **B** WB analysis of p-AKT, P-S6, P-4EBP1, CDCA5 and β-actin levels in ACHN or 786-O with or without CDCA5 knockdown (KD) or CDCA5 overexpression (OE). The expression intensity was compared using arbitrary ratios normalized against β-actin. **C** MTT analysis of the cell proliferation of ACHN NC, and ACHN OE cells with or without Torin1 (100 nM) treatment for 72 h. Data show mean ± SD from three biological replicates

We next investigated the potential mechanisms of CDCA5-mediated ccRCC progression. The activation of CDCA5 has been reported to induce breast tumor progression via PI3K-AKT-mTOR signaling pathway [12, 16]. As well-characterized PI3K-AKT-mTOR effectors, the phosphorylation and activation of AKT, 4E-BP1 and S6 regulates cell survival, proliferation, and growth [17]. Therefore, we expected that CDCA5 induces ccRCC proliferation and migration by activating mTOR pathway. Indeed, we found that CDCA5 knockdown significantly reduced PI3K-AKT-mTOR activity, while its upregulation displayed opposite action in 786-O and ACHN cells (Fig. 5A, B). Importantly, tumors with CDCA5 KD displayed reduced mTOR signaling pathway activity, as evidenced by reduced P-S6 expression (Fig. 4E), confirming the correlation between CDCA5 and mTOR. Torin 1, a mTOR inhibitor, was used to examine whether CDCA5 mediated ccRCC progression via mTOR pathway. Treatment with Torin 1 lead to decreased cell viability of ACHN cells with CDCA5 overexpression in MTT analysis (Fig. 5C). To our surprise, Torin 1 treatment has no effects on migration of CDCA5-overexpression cells (data not shown), indicating that mTOR pathway is not essential for CDCA5-meidated metastasis. In summary, our findings suggest that upregulation of CDCA5 induced mTOR signaling pathway activation, leading to increased cell proliferation in ccRCC.

### CDCA5 interacts with EEF1A1 in ccRCC cells

**Fig. 6:**
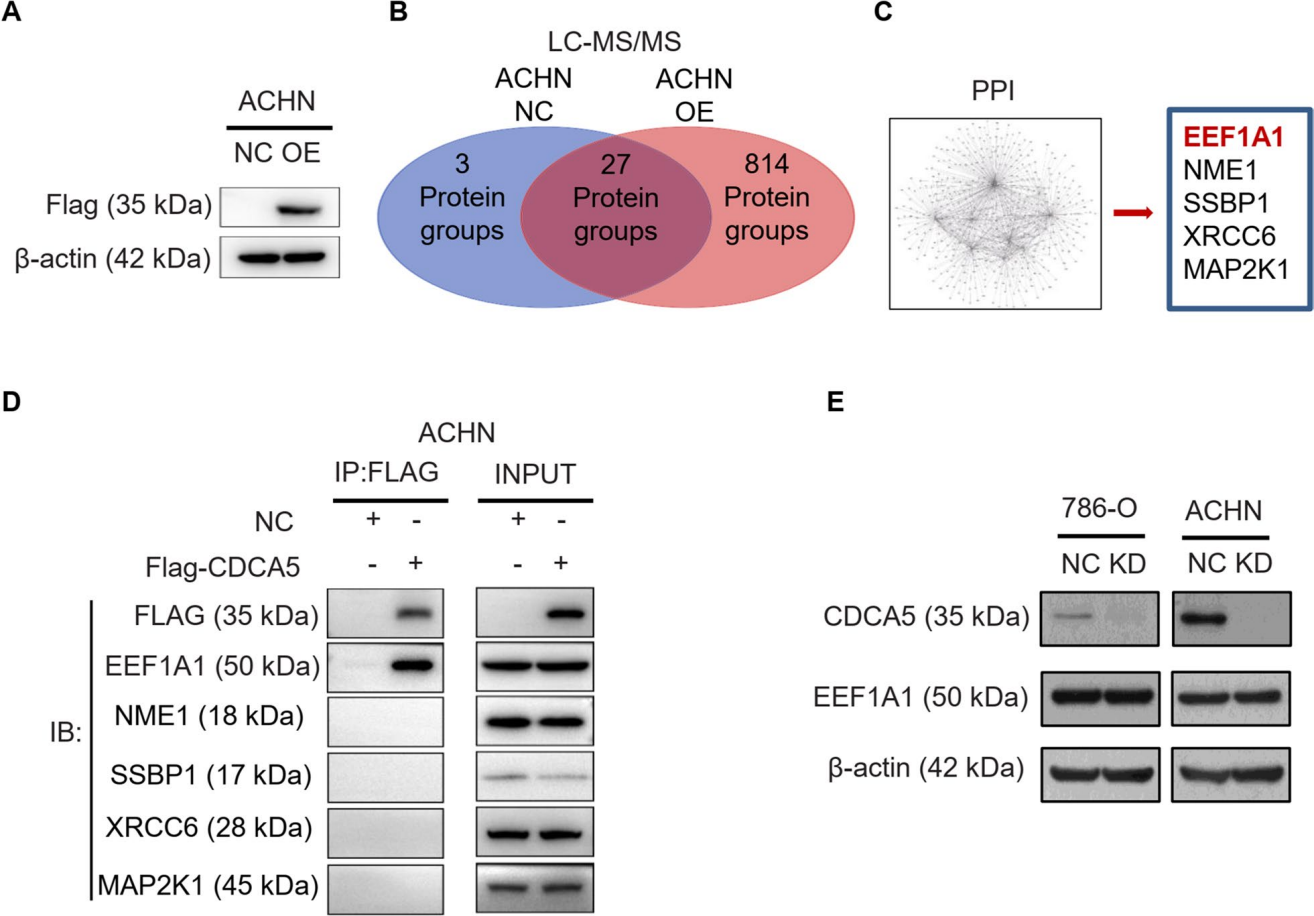
CDCA5 interacts with EEF1A1 in ccRCC cells. **A** WB analysis of CDCA5 expression when overexpressing Flag-CDCA5 (OE) in ACHN cells. **B, C** CDCA5-interacting proteins were identified by LC–MS/MS (**B**), and PPI analysis was used to determine the potential interactors of CDCA5 (**C**). **D, E** Co-IP experiment was used to confirm the interactors of CDCA5 in ccRCC (**D**), and the protein level of EEF1A1 were further evaluated in CDCA5 silenced ccRCC cells. Data show mean ± SD from three biological replicates

The above studies have proved that CDCA5 could activate PI3K-AKT-mTOR signaling pathway promoting tumor growth, but the underlying mechanisms need to be further explored. To this end, we overexpressed FLAG-CDCA5 in ACHN cell line (Fig. 6A, Additional file 4: Fig. S4A). Co-IP assay was performed with Anti-Flag antibody. Using LC–MS/MS experiment with Co-immunoprecipitation protein samples, we identified the potential interacting proteins. PPI analysis results demonstrated that 5 proteins, including EEF1A1, NME1, SSBP1, XRCC6, MAP2K1, may be the candidates interacting proteins of CDCA5 (Fig. 6B, C). To confirm this, EEF1A1 was immunoprecipitated with an anti-FLAG antibody from cell lysates of ACHN cells. Co-IP results showed that EEF1A1, but not other proteins, was co-immunoprecipitated by anti-Flag antibody, indicating that EEF1A1was the interacting protein (Fig. 6D, Additional file 4: Fig. S4B). Furthermore, CDCA5 knockdown did not influence the expression of EEF1A1, suggesting that EEF1A1 is not the downstream target of CDCA5 in ccRCC (Fig. 4E, Additional file 4: Fig. S4C).

### EEF1A1 mediates the impact of CDCA5 on ccRCC cell proliferation and migration via mTOR pathway

**Fig. 7:**
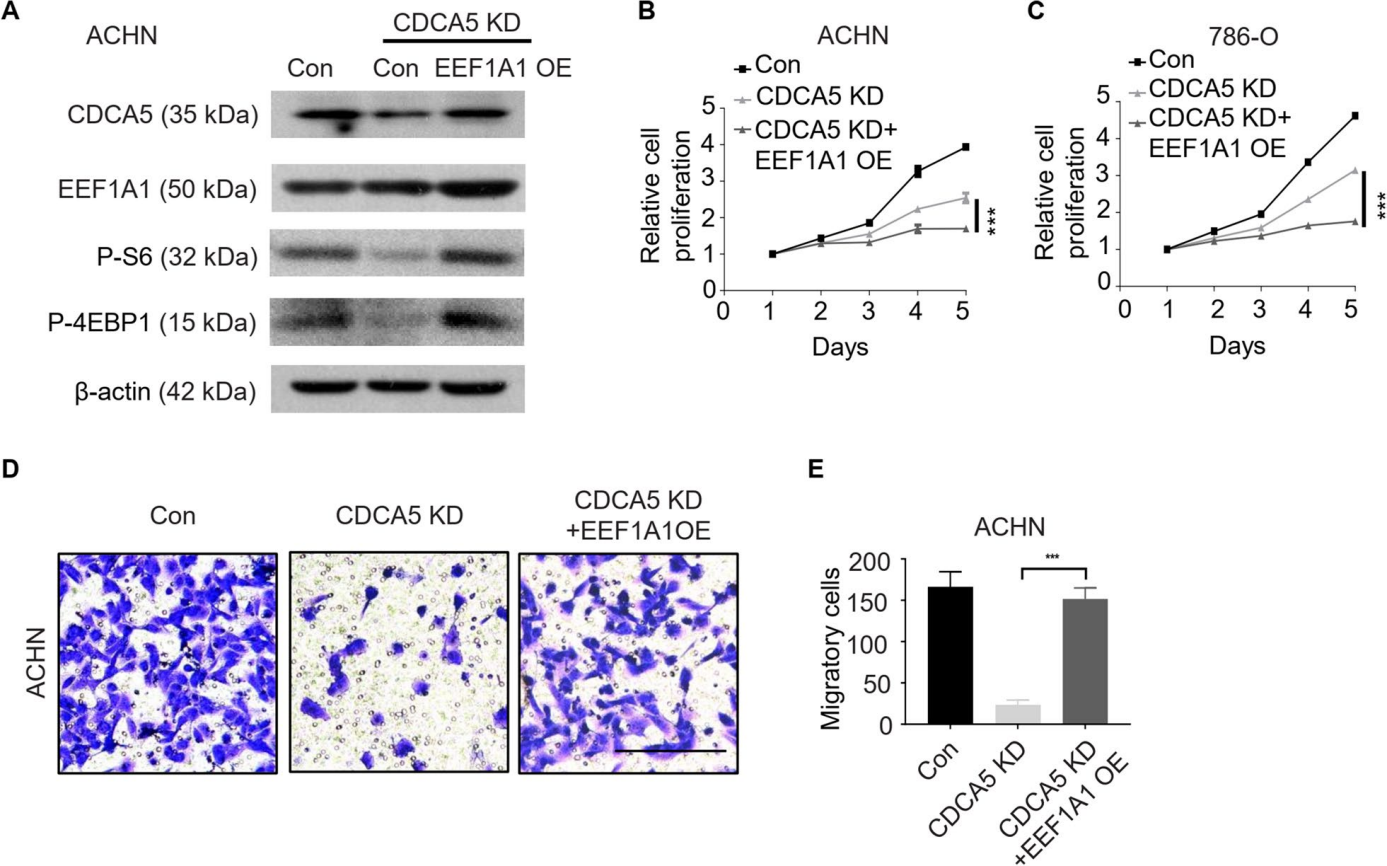
EEF1A1 mediates the impact of CDCA5 on ccRCC cell proliferation and migration via mTOR pathway. **A** EEF1A1 were overexpressed in ACHN cells with CDCA5 knockdown. WB analysis of CDCA5, EEF1A1, P-S6, P-4EBP1 and β-actin levels in ACHN cells, and CDCA5 KD ACHN cells with or without EEF1A1 overexpression (EEF1A1 OE). **B**, **C** Cell proliferation was evaluated by MTT assay in ACHN cells, CDCA5 KD ACHN cells with or without EEF1A1 OE (**B**), as well as 786-O cells, CDCA5 KD 786-O cells with or without EEF1A1 OE (**C**). **D** Cell migration ability was also determined via transwell assay in ACHN cells, CDCA5 KD ACHN cells with or without EEF1A1 OE. **E** Quantification of cell migration in Fig. 7D. ***P < 0.001

Having identified the interaction between CDCA5 and EEF1A1, we wondered whether EEF1A1 had an effect on CDCA5 function. We first overexpressed EEF1A1 in CACA5 knockdown cells (Fig. 7A, Additional file 4: Fig. S4D). MTT and wound healing assays showed that EEF1A1 overexpression reversed the inhibitory effect on the proliferation and migration of ccRCC cells caused by CDCA5 knockdown (Fig. 7B–E). The previous findings have proved that mTOR is essential for CDCA5 function in ccRCC, we thus examine whether the pro-tumor function of CDCA5/EEF1A1 was via mTOR pathway. As expected, upregulation EEF1A1 in CDCA5 knockdown cells induced mTOR activity, as evidenced by increased phosphorylation of key players in mTOR (Fig. 7A), indicating that mTOR may have a role in CDCA5/EEF1A1-mediated ccRCC progression. Taken together, these data strongly suggested that CDCA5 cooperates with EEF1A1 to promote the tumorigenic phenotype in ccRCC.

## Discussion

The mechanism underlying the tumorigenesis of ccRCC is not yet fully understood, and management for ccRCC patients with metastatic disease are currently limited to tyrosine kinase inhibitor (TKI) therapies and immune checkpoint inhibitors therapies [18]. TKI therapies have shown promising antitumor effect and have been approved as the first-line therapy for advanced ccRCC patients [19]. Additionally, immune checkpoint inhibitors, including PD-1/PDL-1 and CTLA-4 antibodies, have emerged as the promising therapeutic options for ccRCC patients [20]. However, none of these agents have a sustained therapeutic effect, mainly due to the acquired drug resistance, tumor heterogeneity and severe side effects [21]. Therefore, there is an urgent need to explore new therapeutic target for advanced ccRCC. In this study, through data-mining across TCGA database and tumor tissues detection, we found that CDCA5 expression is upregulated in ccRCC. Moreover, patients with high CDCA5 expression may exhibit more aggressive disease progression, indicating that CDCA5 may be a potential therapeutic target for RCC clinical therapy.

Cell cycle disruption is a common characteristic of cancers, leading to reduced apoptosis, uncontrolled proliferation, and metastasis in tumor cells [22, 23]. Several cell cycle-associated genes with aberrant functions of are identified in cancer development, presenting potential therapeutic targets for cancer treatment [24, 25]. CDCA5, a member of cell cycle-associated genes, plays a crucial role in preserving genomic integrity by assisting sister chromatid binding and accurate segregation. Functionally, CDCA5 can bind the chromatids during S and G2/M cell cycle phases, and also maintain the stability of DNA strands [5]. A growing body of evidence suggests that CDCA5 is involved in the progression of several types of cancers. However, whether CDCA5 participates in the tumorigenesis and progression of ccRCC has not been thoroughly elucidated. In this study, we reported that CDCA5 is essential for the proliferation and migration ability of ccRCC cells. Silencing of CDCA5 resulted in significant induction of tumor cell apoptosis, in line with increased activity of Caspase3/7. Additionally, upregulation of CDCA5 promoted sunitinib resistance of ccRCC. These findings highlight the potential of targeting as a novel therapeutic approach for ccRCC treatment.

Although CDCA5 is increasingly recognized as an oncogene in various malignancies, the exact mechanisms by which CDCA5 promotes cancer progression remain unclear. Studies in different cancer types have identified specific signaling pathways and regulators involved in CDCA5-mediated functions. For example, Chen H et al. reported that CDCA5 enhances tumor cell proliferation and inhibits cell apoptosis by activating the AKT pathway in hepatocellular carcinoma [26]. Ji J and his colleagues found that MAPK/ERK pathway might be the downstream signal pathway of CDCA5 in prostate cancer [27]. Additionally, CDCA5 cooperates with cyclin-dependent kinase 1 (CDK1) to promote tumor cell proliferation, migration, and invasion abilities in gastric cancer [28]. Consistent with these findings, we found that CDCA5 promotes ccRCC progression via PI3K-mTOR signaling pathway. mTOR inhibition partially reversed CDCA5-mediated proliferation, indicating that alternative mechanisms may be involved in CDCA5 function during ccRCC progression.

Using a combinatory approach of LC–MS/MS analysis and Co-IP confirmation, we identified that EEF1A1 as an interacting protein of CDCA5. Importantly, expression of exogenous EEF1A1 could rescue the suppression of the proliferation and migration ability of ccRCC cells caused by CDCA5 knockdown and activate mTOR signaling pathway. Thus, the interaction between CDCA5 and EEF1A1 may play a crucial role in ccRCC progression. However, the precise regulatory mechanisms of EEF1A1 in regulating mTOR signaling pathway and its essential role in ccRCC remain to be determined.

Of importance, mTOR signaling pathway is a critical regulator of metabolism that participates in the pathogenesis of multiple tumors, such as RCC [29, 30]. Recent studies have connected tumors to various metabolic changes that regulate tumor phenotypes, including cell proliferation and migration [29, 30]. Interestingly, ccRCC is a metabolic disease accompanied with reprogramming of energetic metabolism [31–34], such as abnormal glycolysis [35–37] as shown by impaired mitochondrial bioenergetics and OxPhox, as well as lipid and glutamine metabolism [30, 35, 38–41]. In our study, we found that CDCA5 mediated mTOR signaling was tightly associated with ccRCC proliferation. Therefore, it is worth to investigated whether CDCA5-mTOR axis regulate the cellular and biological process of ccRCC via metabolism in the future study. In addition, mTOR and CDCA5 has been extensively reported to regulate immune cell function and activity as well as tumor immunity [42–44]. Given that tumor microenvironment is tightly associated with tumor progression and therapy [45–49], and ccRCC is a type of classical immune-infiltrated tumor [50–52], we propose that CDCA5-mTOR may also participate tumor immunity and this is deserved to be determined.

In summary, our study demonstrates that CDCA5 functions as a tumor-promoting factor in ccRCC. Mechanically, CDCA5 promotes the tumorigenic phenotype of ccRCC by interacting with EEF1A1 to activate mTOR signaling. These findings suggest that CDCA5 may represent a promising therapeutic target for ccRCC treatment.

### Supplementary Information

**Additional file 1: Figure S1.** (**A**) CDCA5 expression level in multiple tumors and paired normal tissues (Timer database). (**B**) Five-year survival rate of patients with ccRCC according to CDCA5 expression (Timer database). (**C**) RT-qPCR experiments were performed to evaluate CDCA5 mRNA level in 10 pairs of ccRCC tumor tissues and normal tissues. (**D**) Quantification of CDCA5 expression in ccRCC patients in Fig. 1D. (**E**) Quantification of CDCA5 IHC stain score in ccRCC tissues in Fig. 1E. Data show mean ± SD from three biological replicates. **P < 0.01, ***P < 0.001.

**Additional file 2: Figure S2.** (**A**) Quantification of cell apoptosis in Fig. 2F. (**B**) Caspase 3/7 activity was evaluated via Caspase-Glo 3/7 assay. **P < 0.01, ***P < 0.001.

**Additional file 3: Figure S3.** (**A**) Tumor weights were recorded as mean ± SD in different groups, N = 6 for each group. (**B**) Body weight of mice in ACHN NC and KD group. ***P < 0.001.

**Additional file 4: Figure S4.** (**A**) Quantification of Flag-CDCA5 and β-actin expression in ACHN cells in Fig. 6A. (**B**) Quantification of Flag-CDCA5, EEF1A1, NME1, SSBP1, XRCC6, MAP2K1 expression in ACHN cells in Fig. 6D. (**C**) Quantification of CDCA5, EEF1A1 and β-actin expression in ACHN cells in Fig. 6E. (**D**) Quantification of CDCA5, EEF1A1, P-S6, P-4EBP1 and β-actin levels in ACHN cells in Fig. 7A.**P < 0.01, ***P < 0.001.

## Data Availability

The datasets used and analysed during the current study available from the corresponding author on reasonable request.
