# Supplementary figures and images for: CDCA5-EEF1A1 interaction promotes progression of clear cell renal cell carcinoma by regulating mTOR signaling
Source: Cancer Cell Int. 2024 Apr 24;24:147. doi: 10.1186/s12935-024-03330-4 (PMC11044369; doi:10.1186/s12935-024-03330-4)

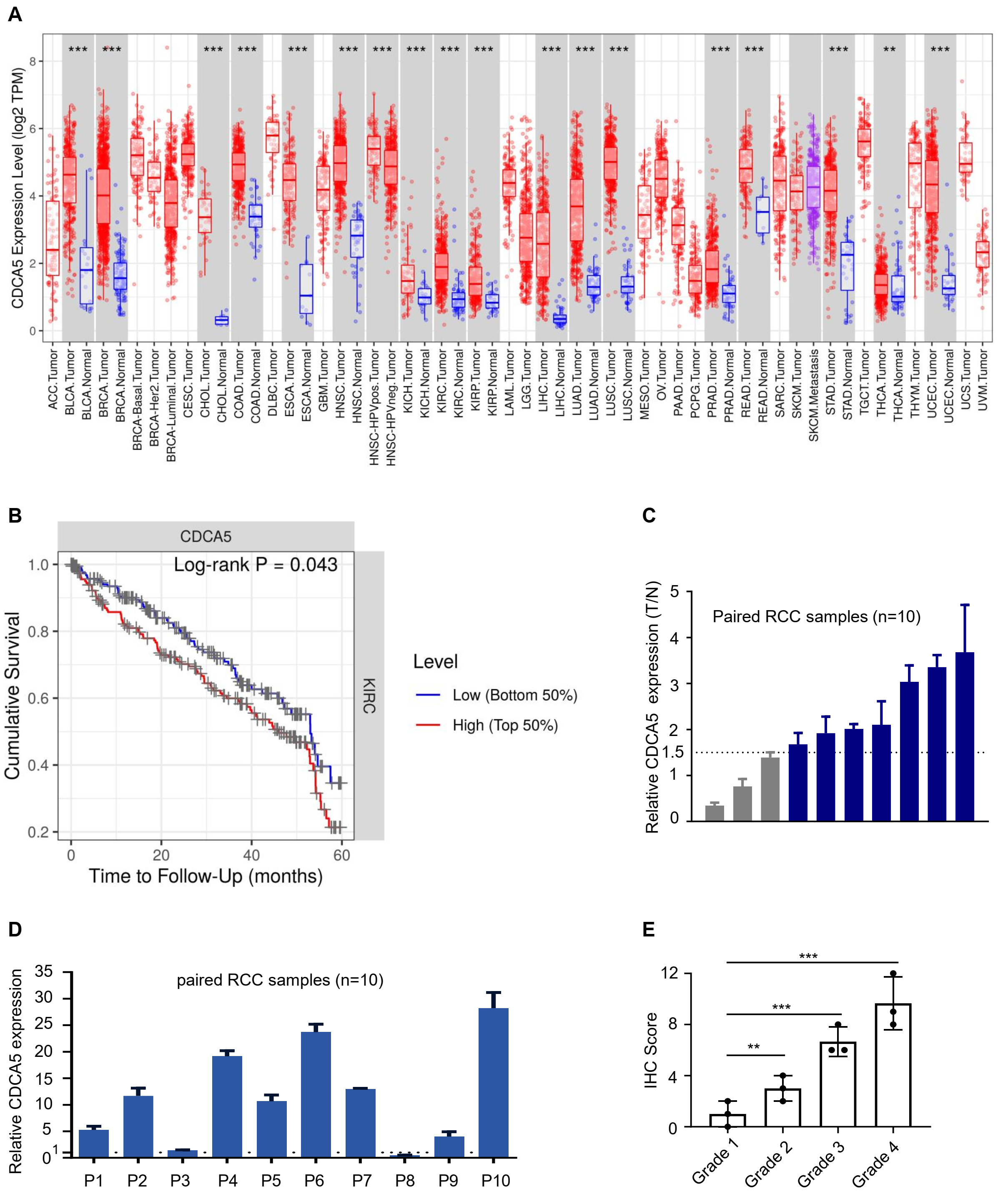

Supplement: Supplementary file 1 — Additional file 1: Figure S1. (A) CDCA5 expression level in multiple tumors and paired normal tissues (Timer database). (B) Five-year survival rate of patients with ccRCC according to CDCA5 expression (Timer database). (C) RT-qPCR experiments were performed to evaluate CDCA5 mRNA level in 10 pairs of ccRCC tumor tissues and normal tissues. (D) Quantification of CDCA5 expression in ccRCC patients in Fig. 1D. (E) Quantification of CDCA5 IHC stain score in ccRCC tissues in Fig. 1E. Data show mean ± SD from three biological replicates. **P < 0.01, ***P < 0.001. [file 12935_2024_3330_MOESM1_ESM.tif]

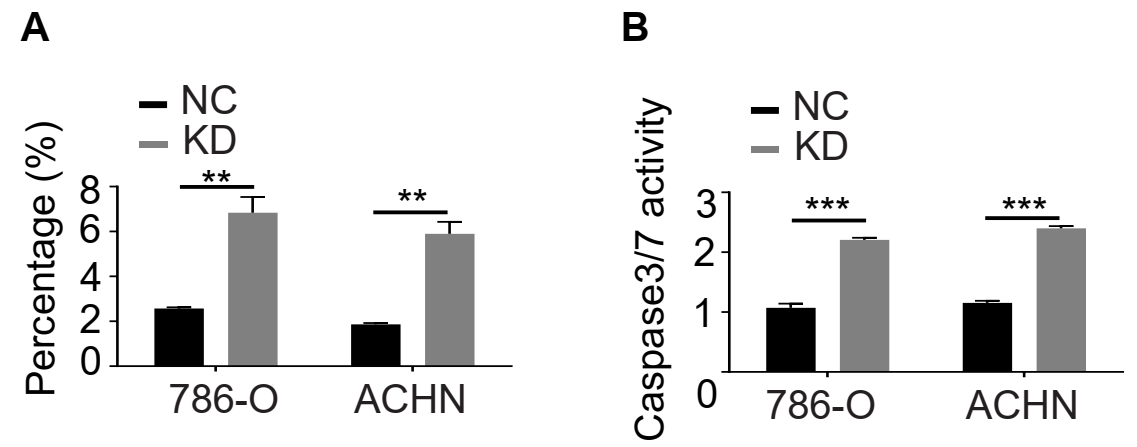

Supplement: Supplementary file 2 — Additional file 2: Figure S2. (A) Quantification of cell apoptosis in Fig. 2F. (B) Caspase 3/7 activity was evaluated via Caspase-Glo 3/7 assay. **P < 0.01, ***P < 0.001. [file 12935_2024_3330_MOESM2_ESM.tif]

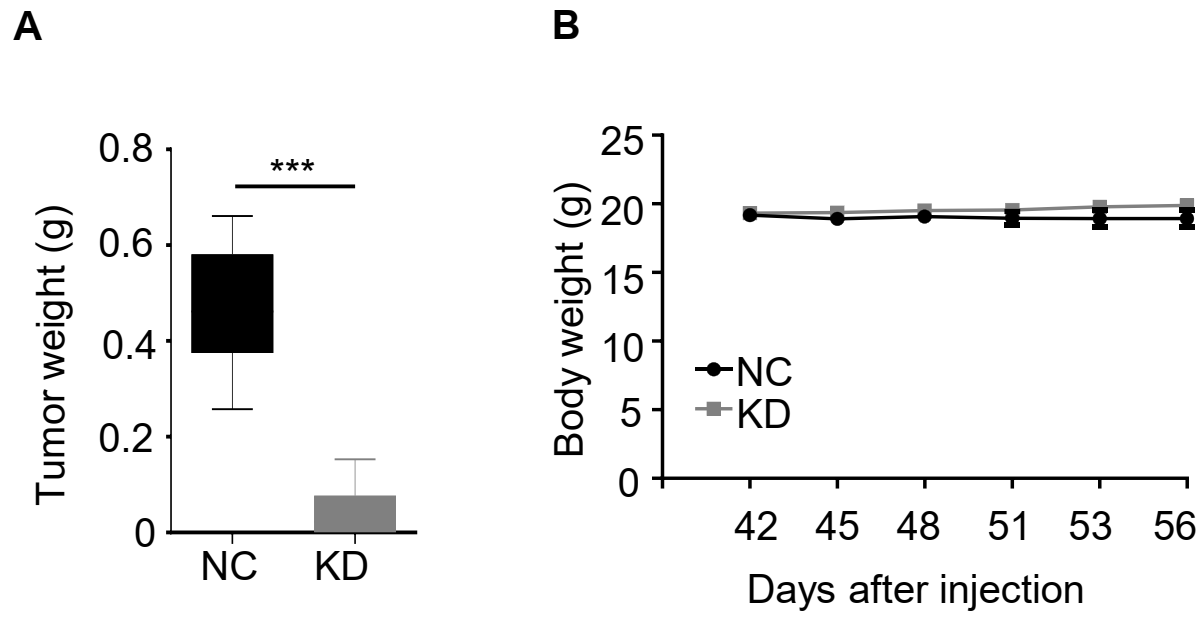

Supplement: Supplementary file 3 — Additional file 3: Figure S3. (A) Tumor weights were recorded as mean ± SD in different groups, N = 6 for each group. (B) Body weight of mice in ACHN NC and KD group. ***P < 0.001. [file 12935_2024_3330_MOESM3_ESM.tif]

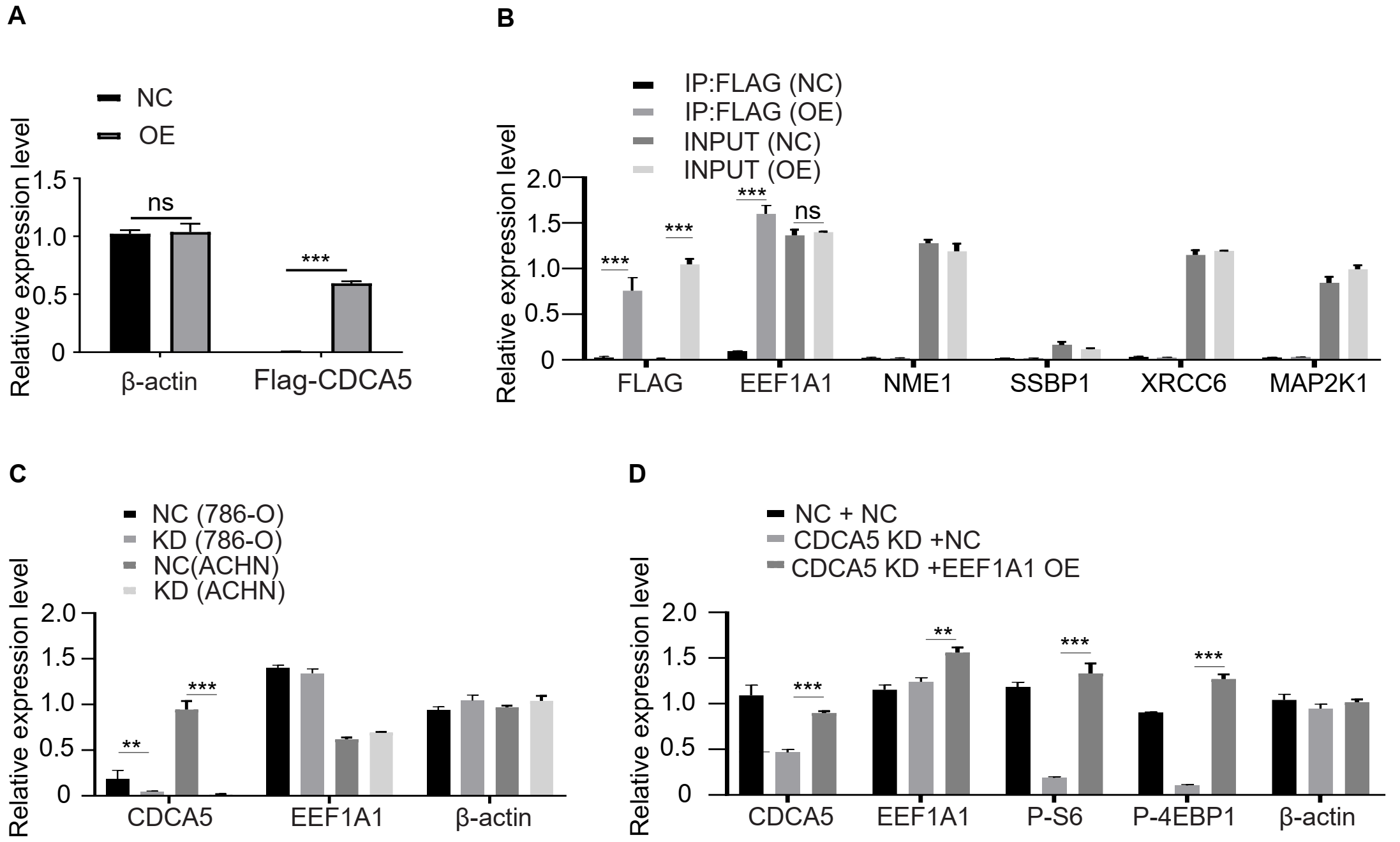

Supplement: Supplementary file 4 — Additional file 4: Figure S4. (A) Quantification of Flag-CDCA5 and β-actin expression in ACHN cells in Fig. 6A. (B) Quantification of Flag-CDCA5, EEF1A1, NME1, SSBP1, XRCC6, MAP2K1 expression in ACHN cells in Fig. 6D. (C) Quantification of CDCA5, EEF1A1 and β-actin expression in ACHN cells in Fig. 6E. (D) Quantification of CDCA5, EEF1A1, P-S6, P-4EBP1 and β-actin levels in ACHN cells in Fig. 7A.**P < 0.01, ***P < 0.001. [file 12935_2024_3330_MOESM4_ESM.tif]
